# Evaluation of multi-inflammatory ındexes in persons living with human immunodeficiency virus: a multicenter study

**DOI:** 10.1590/1806-9282.20250748

**Published:** 2026-03-30

**Authors:** Ahmet Şahin, Tuba Damar Çakırca, Sevil Alkan, Mehmet Çabalak, Fethiye Akgül, Sema Tekin Şahin, Kazım Kıratlı, Mehmet Çelik, Özlem Akay, Selçuk Kaya

**Affiliations:** 1 Gaziantep Islam Science and Technology University, Dr. Ersin Arslan Education and Research Hospital, Faculty of Medicine, Department of Infectious Diseases and Clinical Microbiology – Gaziantep, Turkey.; 2 Şanlıurfa Education and Research Hospital, Department of Infectious Diseases and Clinical Microbiology – Şanlıurfa, Turkey.; 3 Çanakkale Onsekiz Mart University, Faculty of Medicine, Department of Infectious Diseases and Clinical Microbiology – Çanakkale, Turkey.; 4 Hatay Mustafa Kemal University, Tayfur Ata Faculty of Medicine, Department of Infectious Diseases and Clinical Microbiology – Antakya, Turkey.; 5 Batman Education and Research Hospital, Department of Infectious Diseases and Clinical Microbiology – Batman, Turkey.; 6 Alanya Education and Research Hospital, Department of Infectious Diseases and Clinical Microbiology – Antalya, Turkey.; 7 İzmir Atatürk Education and Research Hospital, Department of Infectious Diseases and Clinical Microbiology – İzmir, Turkey.; 8 Harran University, Faculty of Medicine, Department of Infectious Diseases and Clinical Microbiology – Şanlıurfa, Turkey.; 9 Gaziantep Islam Science and Technology University, Faculty of Medicine, Department of Health Sciences-Biostatistics – Gaziantep, Turkey.

**Keywords:** HIV, Inflammation, Treatment

## Abstract

**OBJECTIVE:**

The aim of the study was to evaluate inflammatory markers after 1 year of antiretroviral therapies use in persons living with human immunodeficiency virus.

**METHODS:**

This is a retrospective, observational, and multicenter study. The study included adult, comorbid disease-free patients who received antiretroviral therapies with a diagnosis of human immunodeficiency virus infection. Multi-inflammatory index, multi-inflammatory index obtained by various formulations of complete blood count, C-reactive protein, neutrophil count, lymphocyte count, and platelet count, systemic immune-inflammatory index, neutrophil/lymphocyte ratio, platelet/lymphocyte ratio, C-reactive protein/lymphocyte ratio, cluster of differentiation 4 (CD4) T lymphocyte percentage, and CD4/CD8 ratio data were compared with pretreatment data.

**RESULTS:**

A total of 391 persons living with human immunodeficiency virus, including 241 (61.6%) women, were included in the study. When pretreatment human immunodeficiency virus ribonucleic acid, neutrophil count, lymphocyte count, platelet count, C-reactive protein level, CD4 percentage, CD8 percentage, CD4/CD8 ratio, multi-inflammatory index-1, multi-inflammatory index-2, and systemic immune-inflammatory index data were compared with the 48th week of treatment, a statistically significant difference was found except for neutrophils (p<0.05). When the 48th week post-treatment data were evaluated, human immunodeficiency virus ribonucleic acid, C-reactive protein, multi-inflammatory index-1, multi-inflammatory index-2, and systemic immune-inflammatory index values decreased; lymphocyte, platelet, CD4, CD8, and CD4/CD8 ratio values increased. Among the pretreatment markers, systemic immune-inflammatory index, platelet/lymphocyte ratio, and neutrophil/lymphocyte ratio predicted CD4/CD8 ratio ≤1 or >1 at the end of 48 weeks at the highest rate (60.7, 60, and 59.6%, respectively).

**CONCLUSION:**

Multi-inflammatory markers were found to regress after antiretroviral therapies use in persons living with human immunodeficiency virus. In the long term, these patients should be monitored for the effects of chronic inflammation.

## INTRODUCTION

The human immunodeficiency virus (HIV) was first identified in the United States in 1981 and is the cause of acquired immunodeficiency syndrome (AIDS)^1^. According to World Health Organization (WHO) data, by the end of 2023, the estimated number of people living with HIV worldwide is 39.9 million, 65% of whom are in Africa^2^. HIV in the infected person can be transmitted to a new host through blood, semen, vaginal/rectal fluid from the mucosal membrane; breastfeeding through breast milk, or percutaneous injury. HIV infects cluster of differentiation 4 (CD4+) T lymphocytes, monocytes, macrophages, and dendritic cells. It specifically targets CD4+ T lymphocytes, reducing their numbers and weakening the immune system. When the patient progresses to AIDS, the ability of the immune system to prevent infections decreases, and death is observed due to opportunistic infections^3,4^. Current antiretroviral therapies (ART), which have been used in the treatment of the disease in recent years, reduce viral load when given to infected individuals, improve cellular immunity by increasing CD4+ T lymphocytes, and reduce chronic inflammation. In untreated patients, chronic inflammation can lead to comorbid diseases in the long term. ART initiation has contributed to a decrease in the incidence of HIV-related comorbid diseases such as coronary artery disease, liver and kidney disease, and neurologic diseases due to the decrease in chronic inflammation^5^.

In our multicenter, retrospective, observational study, it was aimed to compare the data of multi-inflammatory index (MII)-1, multi-inflammatory index (MII)-2, systemic immune-inflammatory index (SII), neutrophil/lymphocyte ratio (NLR), platelet/lymphocyte ratio (PLR), C-reactive protein/lymphocyte ratio (CLR), CD4+ T lymphocyte percentage, CD8+ T lymphocyte percentage, and CD4/CD8 ratio, which can be used as inflammatory markers in person living with HIV (PLWH), with the data before ART and at the 48th week of ART.

## METHODS

### Patients and data collection

In this multicenter study, it was planned to include individuals over 18 years of age, without comorbid diseases, who were diagnosed with HIV infection and started antiretroviral therapy between April 2018 and April 2024. It was planned to evaluate various formulations of complete blood count, CRP, neutrophil count, lymphocyte count, and platelet count among the tests performed in patients who were followed up for HIV infection and receiving ART. These formulations are given below^6,7^; NLR=neutrophil-lymphocyte ratio PLR=platelet-lymphocyte ratio

CLR=CRP-lymphocyte ratio

MII-1=NLR×CRP

MII-2=PLR×CRP

SII=(neutrophil×platelet)/lymphocyte

The data of the patients before ART initiation and 48 weeks after ART initiation were retrospectively compared. Patients with HIV infection who did not take ART, those who did not use ART regularly, those with co-infection, and those with comorbid diseases that may cause inflammation, such as collagen tissue disease, autoimmune disease, coronary artery disease, liver and kidney disease, diabetes mellitus, hypertension, malignancy, etc., were not included in the study. Patients with missing data were not included in the study.

### Statistical analysis

Descriptive statistics of the variables used in the study were given as frequency and percentage values for qualitative variables and mean, standard deviation, median, minimum, and maximum values for quantitative variables. In the study, HIV ribonucleic acid (RNA), neutrophil, lymphocyte, platelet count, CRP, CD4 percentage, CD8 percentage, CD4/CD8 ratio, MII-1, MII-2, and SII values of 391 patients before ART and after the 48th week of ART were obtained, and the changes in these data with treatment for HIV patients were investigated. The changes in the data before ART and 48 weeks after ART were analyzed, and their conformity to normal distribution was analyzed by the Kolmogorov-Smirnov test. It was observed that the change variables did not fit the normal distribution (p>0.05). Therefore, the Wilcoxon signed-rank test was used to investigate the changes after treatment. The results of the variable related to the CD4/CD8 ratio at the 48th week of treatment were divided into two groups as 0 (low) for values ≤1 and 1 (high) for values >1. Receiver operating characteristic (ROC) curves were constructed to determine the discrimination of the pretreatment variables MII-1, MII-2, SII, NLR, CLR, and PLR and the appropriate cut-off values that would provide optimum sensitivity and specificity for the variables. To evaluate the diagnostic performance of these test variables, sensitivity, specificity, positive predictive values, negative predictive values, standard error (SE) estimates, areas under the ROC curves (AUCs), and corresponding 95%CIs were calculated. The AUC gives an estimate of the overall accuracy of each variable. An area of 0.50 indicates that the variable adds no information. Spearman’s rank correlation analysis was performed to evaluate the relationship between pretreatment inflammatory indices (SII, NLR, PLR, MII-1, and MII-2) and clinical variables (HIV RNA, CD4, CD4/CD8 ratio, CRP, and PLT). Analyses were performed using the Statistical Package for the Social Sciences 25.0 program, and the significance level was accepted as p<0.05.

### Ethical approval

This study complied with the standards of medical ethics endorsed by decision 437.39.02, dated 02.07.2024, of the Ethics Committee of Gaziantep Islam Science and Technology University.

## RESULTS

In this multicenter, retrospective study, data from a total of 391 PLWH, comprising 150 (38.4%) males and 241 (61.6%) females, were included. The median age of the patients was 34 years (range: 18–83 years). Upon examining the ART regimens received by the patients, 48.8% were on bictegravir+tenofovir alafenamide fumarate/emtricitabine, 29.7% on dolutegravir+tenofovir disoproxil fumarate/emtricitabine, 12% on dolute-gravir+lamivudine, 2% on dolutegravir+lamivudine+abacavir, 5.9% on elvitegravir+cobisistat+tenofovir alafenamide fumarate/emtricitabine, and 1.5% on raltegravir+tenofovir disoproxil fumarate/emtricitabine regimens.

HIV RNA, neutrophil count, lymphocyte count, platelet count, CRP level, CD4 percentage, CD8 percentage, CD4/CD8 ratio, MII-1, MII-2, and SII data of the patients before treatment were compared with the 48th week of treatment. A statistically significant difference was found in all of these parameters except neutrophils (p<0.05). When the 48th week post-treatment data were analyzed, it was observed that HIV RNA, CRP, MII-1, MII-2, and SII values decreased, while lymphocyte, platelet, CD4, CD8, and CD4/CD8 ratio values increased. The parameters compared before and after treatment are shown in Table 1.

**Table 1:** Comparison of pretreatment and post-treatment (Week 48) test variables.

	Pretreatment Median (Min–Max)	Week 48 Median (Min–Max)	p-value
HIV RNA, IU/mL	46,990 (556–942,000,000)	0 (0–4,200)	<0.001^* Ψ^
Neutrophil, ×10^9^/L	3.522 (0.89–13.10)	3.43 (0.98–9.48)	0.564^Ψ^
Lymphocyte, ×10^9^/L	1.550 (0.04–6.46)	2.43 (0.69–9.87)	<0.001^* Ψ^
PLT, × 10^9^/L	218 (42–788)	237 (48–630)	<0.001^* Ψ^
CRP, mg/L	1.30 (0.05–1,081)	1.10 (0–38.80)	<0.001^* Ψ^
CD4 percentage	23 (1–60)	29 (6–67)	<0.001^* Ψ^
CD8 percentage	53 (13–90)	43 (16–91)	<0.001^* Ψ^
CD4/CD8 ratio	0.508 (0.02–1.89)	0.833 (0.08–2.79)	<0.001^* Ψ^
MII-1	5.611 (0.04–1,803.92)	1.736 (0–128.82)	<0.001^* Ψ^
MII-2	318.045 (3.21–163,501.25)	120.396 (0–4,917.54)	<0.001^* Ψ^
SII	531.2 (51.79–19,687.50)	362.424 (14.31–1,844.01)	<0.001^* Ψ^

*p<0.05;

^Ψ^The Wilcoxon test; Min: Minimum; Max: Maximum; HIV RNA: human immunodeficiency virus ribonucleic acid; CRP: C-reactive protein; MII-1: multi-inflammatory index-1; MII-2: multi-inflammatory index-2; SII: systemic immune-inflammatory index; CD: cluster of differentiation; PLT: platelet count. The statistically significant values are presented in bold.

**Figure 1:**
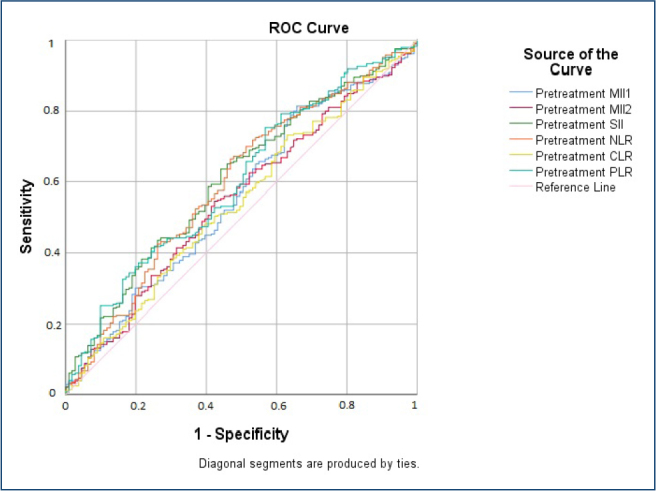
Receiver operating characteristic curves of the six tests for prediction of significant CD4/CD8 ratio (>1) versus CD4/CD8 ration (≤1).

Figure 1 shows the ROC curves of the pretreatment MII-1, MII-2, SII, NLR, CLR, and PLR variables for the correct identification of the qualitative variable in which the CD4/CD8 ratio is 0 (low) when ≤1 and 1 (high) when >1 after 48 weeks of treatment. Table 2 shows the cut-off values, sensitivity, specificity, SE estimates, AUCs, corresponding 95%CIs, and p-values for MII-1, MII-2, SII, NLR, CLR, and PLR variables in the period before ART initiation. According to ROC curve analysis, MII-1 [AUC=0.556 (0.492–0.619), sensitivity=52.1%, specificity=52.3%], MII-2 [AUC=0.550 (0.487–0.612) sensitivity=55.4%, specificity=55.9%], SII [AUC=0.607 (0.546–0.668), sensitivity=58.6%, specificity=59.5%], NLR [AUC=0.596 (0.533–0.659), sensitivity=56.8%, specificity=57.7%], CLR [AUC=0.541 (0.477–0.604), sensitivity=51.4%, specificity=54.1%], and PLR [AUC=0.600 (0.539–0.662), sensitivity=53.2%, specificity=55.9%] with cut-off values of 5.827, 335.804, 587.911, 2.434, 1.526, and 153.868, respectively.

**Table 2:** The sensitivity, specificity, and AUC of the different scores.

Score	Cut-off	Sensitivity (%)	Specificity (%)	AUC (SE)	95%CI		p-value
					Lower limit	Upper limit	
Pretreatment MII-1	5.827	52.1	52.3	0.556 (0.032)	0.492	0.619	0.085
Pretreatment MII-2	335.804	55.4	55.9	0.550 (0.032)	0.487	0.612	0.124
Pretreatment SII	587.911	58.6	59.5	0.607 (0.031)	0.546	0.668	0.001^*^
Pretreatment NLR	2.434	56.8	57.7	0.596 (0.032)	0.533	0.659	0.003^*^
Pretreatment CLR	1.526	51.4	54.1	0.541 (0.032)	0.477	0.604	0.210
Pretreatment PLR	153.868	0.532	0.559	0.600 (0.031)	0.539	0.662	0.002^*^

SE: standard error; AUC: areas under the ROC curve; CI: confidence interval; MII-1: multi-inflammatory index-1; MII-2: multi-inflammatory index-2; SII: systemic immune-inflammatory index; NLR: neutrophil/lymphocyte ratio; CLR: C-reactive protein/lymphocyte ratio; PLR: platelet/lymphocyte ratio.

*p<0.05. The statistically significant values are presented in bold.

Spearman’s rank correlation analysis between pretreatment inflammatory indices (SII, NLR, PLR, MII-1, and MII-2) and clinical variables (HIV RNA, CD4, CD4/CD8 ratio, CRP, and PLT) revealed the following findings: MII-1 showed a moderate positive correlation with CRP (r=0.577; p<0.001), a weak positive correlation with the CD4/CD8 ratio (r=0.292; p<0.001), and a weak negative correlation with PLT (r=-0.109; p=0.032). No significant correlation was observed between MII-1 and HIV RNA or CD4.

## DISCUSSION

HIV infection leads to chronic inflammation. Inflammation may persist despite a decrease in viral load in patients receiving ART and is generally accepted as the cause of comorbid diseases^8^. Most inflammatory biomarkers are not practical due to high cost and difficulty in obtaining them. It is not possible to measure biomarkers in every center. In some studies, as indicators of subclinical inflammation in PLWH, data such as NLR, PLR, and SII, which can be easily analyzed in complete blood count, have been examined^9,10^. In addition to HIV infection, these and similar data have been studied in diseases with active inflammatory activity, such as COVID-19 pneumonia and myocardial infarction^11,12^.

Although CRP is one of the biomarkers used to indicate inflammation, the predictive role of cross-sectional measurement of CRP level in adults with HIV infection remains controversial. This is because CRP level may be transiently elevated during various acute diseases and is therefore nonspecific^13^. ART and CRP levels increase in some patients, decrease in others, and remain the same in others compared to pretreatment levels. In the study by Shivakoti et al. including 205 PLWH, the median CRP levels before ART were found to be similar to the median CRP levels after 48 weeks of ART. However, virologic failure was shown to be more frequent in patients with persistently high CRP levels^14^. In a study conducted in South Africa in which 100 patients were included, a statistically significant decrease in CRP level was observed after 24 weeks of ART. In addition, patients with co-infection had higher CRP levels than those without co-infection (5.2 [3.5–12.6] vs. 3.6 [2.6–7], p=0.019, respectively)^15^. In our study, a decline in CRP levels was also found after ART. However, we think that serial measurements would be more useful instead of CRP levels measured only once.

In some studies, an increase in NLR, PLR, MII, and SII values has been associated with events such as disease activity in systemic lupus erythematosus, increased mortality in patients with COVID-19 pneumonia, and complications in sinus venous thrombosis^16,17,18^. In our study, a statistically significant decrease was observed in MII-1, MII-2, and SII data after 48 weeks of ART. However, we found a decrease in these inflammatory markers with ART use in PLWH. On the other hand, we cannot predict whether comorbid diseases that may accompany it in the long term will decrease in the long term. We believe that patients in whom these rates do not decrease despite effective, up-to-date ART should be followed closely for the development of comorbidities in the following years.

In PLWH, ART use is associated with a decrease in viral load and an increase in CD4 T lymphocyte count and CD4/CD8 ratio. It is recognized that a low CD4/CD8 ratio is associated with an increased risk of serious non-AIDS events. Many clinicians now believe that the CD4/CD8 ratio can be helpful in HIV surveillance. However, some recent studies have not provided reliable and uniform results on the ability of the CD4/CD8 ratio to predict adverse outcomes^19^. It was found that the CD4/CD8 ratio measured in the 2nd year of 5,133 patients receiving ART did not predict non-AIDS events in the following 5 years^20^. In a study involving 4,625 PLWH, it was found that the CD4/CD8 <0.3 measured after 2 years of ART use predicted the development of cardiovascular events and malignancy in the next 5 years^21^. In another study including 557 patients, it was thought that CD4/CD8 ratio could predict non-AIDS events, but dynamic measurements should be made^22^. In our study, significant increases were found in CD4 T lymphocyte count and CD4/CD8 ratio after 48 weeks of ART use compared to pretreatment.

### Limitations

Our study had some limitations. Since only HIV-infected patients without comorbidity were included in this study, we do not know whether the results will change in patients with comorbidity. Our other limitation was that we did not have long-term data on the development of complications and comorbidities despite the regression in inflammatory markers in patients with follow-up. Third, we did not include the number of years the patients had been living with HIV in the study. Because the duration of HIV infection may affect the inflammatory response and the results of our study.

## CONCLUSION

In conclusion, in our study, we found that CRP, MII-1, MII-2, SII, NLR, and PLR, which are used as inflammatory markers in many non-HIV studies, decreased in PLWH after ART initiation. However, we acknowledge that the results of these markers are modest. Considering that inflammatory biomarkers are expensive, difficult to obtain, and challenging to analyze, we believe that inexpensive inflammatory markers could be used to monitor PLWH living in countries with high patient loads and limited resources over the long term.

## Data Availability

The datasets generated and/or analyzed during the current study are available from the corresponding author upon reasonable request.
